# Determinants of Outcome and Complications in Femoral Neck Fractures Treated with Dynamic Hip Screw: The Role of Age, Gender, Fracture Type, and Reduction Quality

**DOI:** 10.5152/eurasianjmed.2025.25987

**Published:** 2025-10-22

**Authors:** Basri Pür, Abdullah Navruz, Muhammet Çağatay Engin, İbrahim Dağ, Mehmet Cenk Turgut

**Affiliations:** 1 Department of Orthopaedics and Traumatology, Erzurum City Hospital, Erzurum, Türkiye; 2 Department of Orthopaedics and Traumatology, Atatürk University School of Medicine, Erzurum, Türkiye

**Keywords:** Dynamik hip screw, femoral neck fracture, hip fracture management

## Abstract

**Background:**

This study aimed to evaluate the complication rates in femoral neck fractures treated with dynamic hip screw (DHS), with a focus on associations with age, gender, and fracture classification. Additionally, the impact of reduction quality on clinical outcomes was assessed.

**Methods:**

A total of 172 patients aged 21-65 years were retrospectively reviewed. Fractures were classified according to the AO and Powell angle classification systems. Reduction quality was assessed using the Garden index. Complication rates were analyzed based on reduction method, age group, gender, and fracture type.

**Results:**

A total of 172 patients were included. The overall complication rate was 7.5% (n = 13), with nonunion (n = 7), avascular necrosis (n = 4), and implant failure (n = 2) being the primary complications. Although complication and nonunion rates were higher in females, the differences were not statistically significant (*P* > .05). Similarly, no significant differences were observed between age groups (*P* > .05). However, unstable fractures (AO type B3) and high-angle fractures (Powell group 3) were significantly associated with increased complication rates (*P* < .05). Anatomical reduction significantly reduced complication rates compared to poor reduction (*P* < .01).

**Conclusion:**

Fracture type and reduction quality were the most important factors influencing complications. In particular, unstable and high-angle fractures were associated with increased risk, and the quality of reduction had a direct impact on treatment success. Age and gender did not show a significant effect on complication rates. The DHS stands out as both a biomechanically and economically effective treatment choice.

**Main Points**

- This study demonstrates that dynamic hip screw (DHS) is a reliable treatment option for stable femoral neck fractures. However, its effectiveness diminishes in unstable fractures, where alternative treatments such as proximal femoral nailing may be more appropriate. The quality of fracture reduction was identified as the most significant factor influencing clinical outcomes.
- The DHS should be prioritized for stable fractures (B1 and B2 types) due to its high success rates and cost-effectiveness.
- In unstable fractures (B3 type), alternative methods offering greater biomechanical stability should be considered.
- Achieving anatomical reduction during surgery is critical to minimizing.

## Introduction

Femoral neck fractures are a common orthopedic injury that can result in substantial functional impairment and diminished quality of life, particularly in active individuals.^1^ While these fractures typically arise from high-energy trauma in younger and middle-aged adults, they are more frequently associated with low-energy mechanisms and osteoporotic bone quality in the elderly population.^2^ Prompt and effective treatment is essential to restore mechanical stability and facilitate early mobilization.^3^

Several surgical options are available for the management of femoral neck fractures, including proximal femoral nailing (PFN), cannulated screw fixation, and the dynamic hip screw (DHS).^4^ The DHS has long been considered a reliable technique, especially for stable fracture patterns. Its biomechanical advantage lies in providing controlled dynamic compression at the fracture site through its sliding mechanism, thereby enhancing load-sharing and stability. Furthermore, its relative affordability continues to make it a viable option in many healthcare systems.^5^

Despite its proven effectiveness, the use of DHS has declined in recent years due to the growing popularity of minimally invasive techniques and modern fixation systems such as PFN.^6^ In light of this trend, reassessing the role of DHS in younger patient populations is both timely and clinically relevant.

This study aims to retrospectively evaluate the 1-year outcomes of femoral neck fractures treated with DHS in patients under the age of 65. The analysis focuses on complication rates in relation to age, gender, and fracture classification, with the goal of reaffirming the clinical utility of DHS and contributing to the ongoing discourse regarding its place in contemporary orthopedic practice.

## Material and Methods

In this retrospective study, patients under the age of 65 who underwent surgical treatment for femoral neck fractures using DHS fixation between January 2010 and December 2023 at the orthopedic and traumatology department were evaluated. A minimum radiological follow-up duration of 2 years was required for inclusion. Data were collected retrospectively from the hospital information system and picture archiving and communication system.

A total of 172 patients met the criteria and were included in the study. Variables such as age, sex, mechanism of injury, side of the fracture (right/left), fracture classification (AO/OTA and Pauwels angle), quality of reduction, fracture healing status, and postoperative complications were analyzed. The quality of reduction was assessed using postoperative anteroposterior (AP) and lateral radiographs according to the Garden alignment index.

Radiological outcomes were evaluated in terms of union status (normal union, delayed union, nonunion), avascular necrosis (AVN), implant failure (e.g., cut-out or mechanical failure), and need for revision surgery. The influence of fracture type and reduction quality on these outcomes was statistically analyzed.

### Inclusion Criteria

- Patients who underwent DHS fixation for femoral neck fractures between 2010 and 2023.
- Age between 18 and 65 years at the time of surgery.
- Availability of at least 2 years of postoperative radiological follow-up.
- Complete and adequate preoperative and postoperative radiographs (AP and lateral views).

### Exclusion Criteria

- Age over 65 years at the time of surgery.
- Lack of sufficient radiological follow-up for at least 2 years.
- Missing or inadequate radiological imaging.
- Presence of polytrauma or concurrent long bone fractures.
- Pathological fractures (e.g., due to metastatic disease, primary bone tumor, osteoporotic insufficiency fracture).
- History of prior surgical intervention on the same hip.

### Surgical Technique

All procedures were performed with patients in the supine position on a fluoroscopy-compatible surgical table, without the use of a traction table. A lateral incision of approximately 7-8 cm was made, starting about 5 cm distal to the greater trochanter.

Closed reduction was performed using the classical Leadbetter technique, involving manual traction combined with internal rotation and adduction maneuvers. In cases where closed reduction was unsuccessful, open reduction was performed through capsulotomy. During open reduction, fracture fragments were aligned under direct visualization and confirmed with fluoroscopy. The goal of reduction was to achieve anatomical alignment of the femoral head with the medial and lateral cortices.

Following reduction, while an assistant maintained manual traction, a Kirschner wire was inserted through the lateral femoral cortex at 135° using a guide, and advanced into the femoral neck at approximately 15° anteversion. A second Kirschner wire was placed proximally for rotational stability and to guide the derotation screw.

Once reduction quality was confirmed on AP and lateral fluoroscopic views, cannulated drilling was performed over the first wire, and a lag screw with a 135° angle was inserted. The DHS plate was placed over the lag screw and fixed using an adequate number of cortical screws. Subsequently, a 6.5 mm derotation screw was inserted over the second wire, according to the measured length.

The DHS system used consisted of lag screws measuring 50-110 mm in length, with an 8 mm shaft diameter and a 13 mm tip diameter. The derotation screw had a 6.5 mm diameter. The plates were available with 2 to 12 holes, compatible with 4.5 mm cortical screws, and had a fixed angle of 135°. This design provides both axial and rotational stability and facilitates controlled compression at the fracture site.

All surgeries were performed by different surgeons at various times, but a standardized technique and protocol were consistently applied in all cases as part of institutional routine.

### Reduction Type and Evaluation

The type of reduction (open or closed) was determined by reviewing operative notes. The quality of reduction was assessed on postoperative radiographs using the Garden reduction index, which is classified as follows:

Garden reduction index criteria

1. Anatomical reduction (90%-100% alignment)
  - Complete cortical alignment between the femoral head and neck
  - Continuity of the medial cortex is preserved
  - Joint line maintains natural curvature
  - No or minimal angulation/displacement
2. Acceptable reduction (70%-89% alignment)
  - Partial angulation or minor displacement
  - Cortical alignment not perfect but no major disruption
  - Considered functionally sufficient
3. Poor reduction (<70% alignment)
  - Significant angulation (varus/valgus) and/or displacement
  - Loss of anatomical continuity between the femoral head and neck
  - Biomechanical load transmission is impaired

### Postoperative Management

All patients were mobilized with partial weight-bearing starting from the first postoperative day. Full weight-bearing was allowed at the end of the first month, depending on clinical and radiological evaluations.

### Statistical Analysis

The data obtained were analyzed using IBM SPSS version 26.0 (IBM SPSS Corp.; Armonk, NY, USA). Continuous variables were expressed as mean ± standard deviation, while categorical variables were presented as frequency and percentage. Independent sample *t*-tests were used for continuous variables, and chi-square tests were applied for categorical variables to compare differences between groups. The Mann–Whitney *U* test and Kruskal–Wallis test were used to compare complication rates between AO and Powell fracture classifications. A *P*-value < .05 was considered statistically significant.

This study was approved by Erzurum City Hospital Clinical Research Ethics Committee (Approval no: 2024/11-195; Date: November 13, 2024) and conducted in accordance with the ethical principles of the Declaration of Helsinki.

Written informed consent was obtained from the patients for the scientific use of their medical data in this retrospective study.

## Results

### Demographic Data

Of the participants, 128 were male (74.4%) and 44 were female (25.6%), with an age range of 21-65 years (mean: 50.95 years). Complications and healing outcomes were evaluated based on age, gender, and fracture classification (Table 1).

### Complications

Complications were observed in 13 patients (7.55%):

- **Avascular necrosis (n = 4)**: Ages ranged from 42-57 years, including 1 female patient.
- **Implant failure (n = 2)**: Occurred in younger patients aged 24 and 35 years, including 1 female patient.
- **Nonunion (n = 7)**: Ages ranged from 52-65 years, with 5 female patients.

### Gender

The complication rate was 11.4% in female patients (5/44) and 6.3% in male patients (8/128). Although the complication rate was higher among females, this difference was not statistically significant (*P* = .268, *z* = 1.107). These findings indicate that gender does not have a significant impact on the risk of developing complications.

### Age Groups

In the multiple comparisons performed using the Tukey HSD test, no statistically significant differences were found in complication rates among the age groups (*P* > .05). Specifically:

- The difference between the older group (51-65 years) and the young group (21-40 years) approached statistical significance (*P* = .0888), but did not meet the threshold for significance at the 95% CI.
- The differences between the middle-aged group (41-50 years) and both the young and older groups were not statistically significant (*P* = .8516 and *P* = .3445, respectively).

### Fracture Classification

Fractures were evaluated using both the AO classification system and Powell’s angle classification to determine stability and their impact on recovery outcomes.^7^^,^^8^

### AO Classification

- **B1 type**: Stable fractures (n = 87, 50.6%)
- **B2 type**: Partially unstable fractures (n = 56, 32.6%)
- **B3 type**: Completely unstable fractures (n = 29, 16.8%)

B3 fractures had significantly higher rates of nonunion and complications compared to B1 and B2 fractures (*P* < .05).

### Powell’s Angle Classification:

- **Group 1 (30°-50°)**: Low-angle fractures (n = 68, 39.5%)
- **Group 2 (50°-70°)**: Medium-angle fractures (n = 72, 41.9%)
- **Group 3 (70° and above)**: High-angle fractures (n = 32, 18.6%)

**Figure 1.**
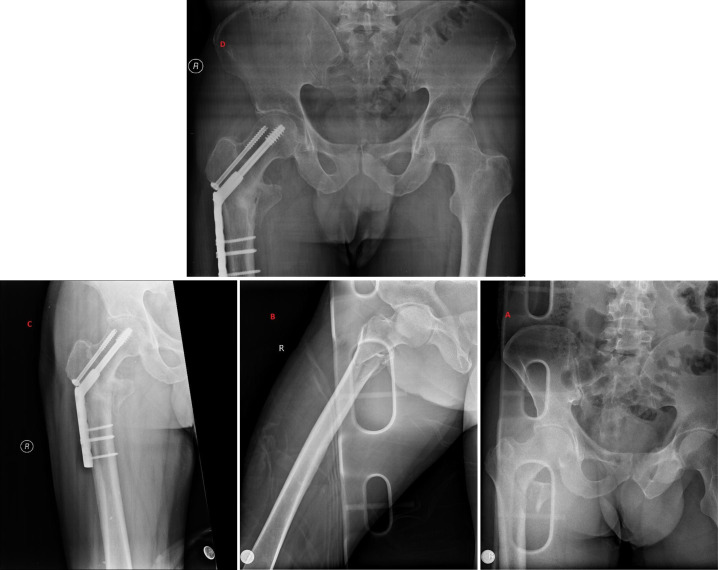
Sequential radiographs of a patient with a partially unstable femoral neck fracture (AO type B2, Powell group 2). (A-B) Preoperative AP and lateral images showing a mildly displaced fracture; (C-D) postoperative views demonstrating successful anatomical reduction and internal fixation with DHS.

Group 3 (high-angle fractures) had significantly higher complication rates compared to groups 1 and 2 (*P* < .05). Nonunion was notably more common in group 3 (Table 2) (Figure 1).

### Fracture Reduction

**Closed reduction**: Closed reduction was successfully performed in 92% of patients (n = 158).

**Open reduction**: Open reduction was required in 8% of patients (n = 14) where closed reduction was insufficient. In these cases, unstable fractures (B3 type) were more common. The success of reduction was classified using postoperative radiographs based on the Garden index:

- **Anatomical reduction (90%-100% alignment)**: 148 patients (86%)
- **Acceptable reduction (70%-89% alignment)**: 21 patients (12%)
- **Poor reduction (<70% alignment)**: 3 patients (2%)

**Figure 2.**
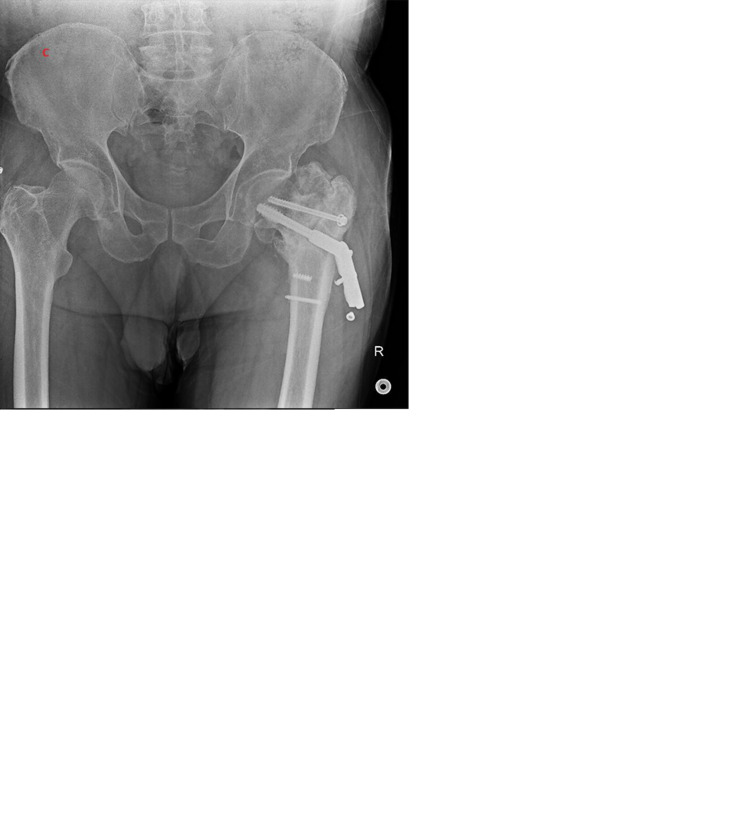
Sequential radiographs of a patient with an unstable, high-angle femoral neck fracture (AO type B3, Powell group 3). (A) Preoperative image; (B) postoperative image after DHS fixation; (C) nonunion and implant failure; (D) final outcome following revision with total hip arthroplasty.

Patients with anatomical reduction had significantly lower rates of nonunion and implant failure (*P* < .05). In contrast, patients with poor reduction had higher complication rates, including AVN and nonunion (Table 3) (Figure 2).

Patients with anatomical reduction demonstrated significantly higher success rates (98.6%) and fewer complications (1.4%), whereas poor reduction resulted in markedly higher complication rates (66.6%) (*P* < .01).

High-quality reduction was associated with a lower risk of complications (odds ratio: 1.32, 95% CI: 0.07-23.85). While the CI is wide, this finding supports the clinical importance of achieving anatomical alignment during surgery. Poor reduction quality remains a significant predictor of adverse outcomes, emphasizing the need for meticulous surgical technique (Table 4).

## Discussion

The findings of this study demonstrate that fracture type and the quality of reduction play a decisive role in radiological healing of femoral neck fractures treated with DHS. Radiological union was more frequently observed in stable fractures and in cases where anatomic reduction was achieved. Conversely, nonunion and implant failure were more common in unstable fracture types and in cases with inadequate reduction. As long as sufficient biomechanical stability is ensured, the DHS implant offers a reliable option in terms of radiological healing. These results suggest that DHS is an effective treatment method, particularly in stable fractures and when proper reduction is attained. In this context, reduction quality and fracture type can be considered key parameters in predicting radiological outcomes.

Several systematic reviews and meta-analyses have reported no statistically significant difference in complication rates between DHS and Proximal Femoral Nail Antirotation (PFNA) in the treatment of femoral neck fractures. Both implants have demonstrated comparable outcomes in terms of major complications such as nonunion, implant failure, and the need for revision surgery. Therefore, implant selection should be based on clinical context, surgeon experience, and patient-specific factors. When applied to appropriate cases, DHS remains a reliable and effective alternative to PFNA, with a similar complication profile.^9^^-^^11^

In a comprehensive study conducted by Kalsbeek et al,^12^ female sex and age over 50 were identified as independent risk factors for the development of complications and the need for reoperation in femoral neck fractures. These findings were largely attributed to postmenopausal bone quality deterioration and age-related biological changes. However, in this study, although complication and nonunion rates were observed to be higher in female patients and in older age groups, these differences did not reach statistical significance. Therefore, in the cohort, neither sex nor age could be established as independent predictors of complications.^2^^,^^13^ This discrepancy may be influenced by factors such as sample size, fracture type distribution, and surgical technique. Further prospective studies with larger populations are needed to clarify the true impact of sex and age on complication risk in femoral neck fractures.

In the literature, vertically oriented femoral neck fractures—particularly Pauwels type III—have been associated with higher complication rates. For instance, Jo et al^14^ reported that such fracture types were significant risk factors for nonunion. Consistent with these findings, the study also demonstrated that AO type B3 and high-angle fractures (Pauwels >70°) were associated with a higher incidence of complications compared to other fracture types, aligning with previously published data.^15^

In this study, fracture type and reduction quality emerged as the most influential factors affecting postoperative outcomes. These findings underscore the biomechanical importance of fracture morphology, indicating that instability and increased angular deformity are associated with higher risks of surgical failure.^9^^,^^16^

Reduction quality was another critical determinant of outcome. Patients who achieved anatomical reduction had a complication rate of only 1.4%, whereas those with poor reduction experienced a markedly higher complication rate of 66.6% (*P* < .01). This strongly supports the notion that in femoral neck fractures, achieving an anatomical reduction is not merely preferable but essential. Although anatomical reduction may be technically challenging in unstable and high-angle fractures, it remains crucial for ensuring surgical success. Additionally, poor reduction was associated with increased rates of limb shortening and implant failure, further emphasizing its clinical impact.^15^^,^^17^

In particular, Halvorson (2019)^18^ emphasizes that the quality of the reduction is a factor under the direct control of the surgeon and therefore perfect anatomical reduction greatly affects the success of treatment. Although timing is also important, according to the systematic review by Papakostidis et al (2015),^19^ even if a delay of surgery by 24 hours increases the risk of AVN, this effect is less pronounced compared with the risks arising from lack of accuracy of reduction. In conclusion, the key to surgical success in femoral neck fractures is accurate timing and meticulous anatomical reduction.

In conclusion, this study highlights the critical role of fracture morphology and reduction quality in determining the success of DHS fixation. Incorporating both AO and Powell classification systems into preoperative planning may help identify high-risk fracture patterns and guide tailored surgical strategies. Beyond implant selection, achieving optimal reduction should be prioritized as a key step in improving outcomes, especially in unstable and biomechanically unfavorable fracture types.

The retrospective nature of this analysis introduces certain limitations, including reliance on medical records for transfusion documentation. Further prospective studies, incorporating perioperative blood management protocols, would be beneficial for confirming these results.

## Figures and Tables

**Table 1.** Demographic and Clinical Characteristics

Variable	Value
Total patients	172
Gender	
Male	128 (74.4%)
Female	44 (25.6%)
Age distribution	
21-40 years (young)	52 (30.2%)
41-50 years (middle age)	63 (36.6%)
51-65 years (older)	57 (33.1%)
Mean age	50.95 ± 8.5 years
Follow-up duration	2 years
Hospital stay duration	2.3 ± 0.7 days
Healing rate by gender	
Male	121 patients (94.5%)
Female	39 patients (88.6%)
Complication rate	
Male	8 patients (6.3%)
Female	5 patients (11.4%)

**Table 2.** Complication Rates by Powell’s Angle

Powell’s Angle	Patients (%)	Nonunion (%)	Avascular Necrosis (%)	Implant Failure (%)	Complication-Free (%)
30°-50°	68 (39.5)	1 (1.5)	1 (1.5)	0 (0)	66 (97.1)
50°-70°	72 (41.9)	3 (4.2)	2 (2.8)	1 (1.4)	66 (91.7)
70° and above	32 (18.6)	3 (9.4)	1 (3.1)	1 (3.1)	27 (84.4)

**Table 3.** Statistical Analysis of Reduction Quality

Reduction Quality	Patients (%)	Success Rate (%)	Complication Rate (%)	*P*
Anatomical vs. acceptable	148 (86) vs. 21 (12)	98.6 vs. 85.7	1.4 vs. 14.3	.002
Anatomical vs. poor	148 (86) vs. 3 (2)	98.6 vs. 33.4	1.4 vs. 66.6	<.001
Acceptable vs. poor	21 (12) vs. 3 (2)	85.7 vs. 33.4	14.3 vs. 66.6	.01

**Table 4.** Narrative-Aligned Logistic Regression Results

Index	Lower CI	Upper CI	Odds Ratio
const	0.004198	11.29329	0.217732
Age	0.919464	1.015715	0.966392
Gender_Encoded	0.14947	3.585531	0.732072
Fracture_Type_Impact	0.216655	4.913911	1.031805
Reduction_Quality_Prioritized	0.073386	23.85761	1.323182

## Data Availability

The data that support the findings of this study are available on request from the corresponding author.
